# Early sedation using ciprofol for intensive care unit patients requiring mechanical ventilation: a pooled post-hoc analysis of data from phase 2 and phase 3 trials

**DOI:** 10.1186/s13613-024-01390-3

**Published:** 2024-10-26

**Authors:** Yongjun Liu, Lingyun Zuo, Xiaoyun Li, Yao Nie, Chuanxi Chen, Ning Liu, Minying Chen, Jianfeng Wu, Xiangdong Guan

**Affiliations:** 1 Department of Critical Care Medicine, The First Affiliated Hospital, Sun Yat-sen University, Guangzhou, 510080 China; 2 Guangdong Clinical Research Center for Critical Care Medicine, No. 58 Zhongshan 2nd Road, Guangzhou, 510080 China

**Keywords:** Ciprofol, Pooled analysis, Early sedation, Intensive care unit, Mechanical ventilation

## Abstract

**Background:**

Ciprofol was approved for use in intensive care unit (ICU) patients requiring sedation during mechanical ventilation in July 2022. A pooled post-hoc analysis of phase 2 and phase 3 trials was conducted primarily to explore hypotension-free outcome in ICU patients who required mechanical ventilation and achieved the target light sedation goal at an early stage after being sedated with ciprofol or propofol.

**Methods:**

All eligible ICU patients who were expected to require sedation for 6–24 h were randomly assigned in a 2:1 ratio to either a ciprofol or propofol group. Ciprofol or propofol was initially infused at loading doses of 0.5 or 1.0 mg/kg followed by maintenance doses of 0.3 or 1.5 mg/kg/h. Ciprofol or propofol dosages were adjusted up or down at rates of 0.05–0.10 mg/kg/h or 0.25–0.50 mg/kg/h, respectively, to achieve the target light sedation (a Richmond Agitation-Sedation Scale of -2 to + 1). The primary post-hoc outcome was the hypotension-free rate in patients who had achieved the target sedation goal after 30-min administration of ciprofol or propofol.

**Results:**

In total, 174 patients were enrolled for pooled post-hoc analysis, of whom 116 and 58 were assigned to the ciprofol and propofol groups, respectively. The hypotension-free rate was significantly higher in patients who achieved the target sedation goal after 30-min administration of ciprofol (93.0% vs. 81.0%, *P* = 0.018), and especially in the subgroups of males and patients aged < 65 years. Multivariable analysis revealed that ciprofol treatment, a younger age and lower baseline body mass index were independent favorable predictors for a higher hypotension-free rate in patients who achieved the target sedation goal after 30-min of drug administration. Moreover, hypotension-free patients who reached the target sedation level after 30 min had a more favorable short-term prognosis including a lower incidence of drug-related treatment-emergent adverse events, shorter time to extubation and fewer dose adjustments of ciprofol or propofol (all *P* < 0.05).

**Conclusion:**

ICU patients undergoing mechanical ventilation and sedated with ciprofol had significantly lower rate of hypotension during the early phase of achieving light sedation during a 6–24 h period, leading to a more favorable short-term prognosis (within 24 h).

**Trial registration:**

Phase 2 trial (clinicaltrials.gov, NCT04147416. Registered November 1, 2019, https://classic.clinicaltrials.gov/ct2/show/NCT04147416) and phase 3 trial (clinicaltrials.gov, NCT04620031. Registered November 6, 2020, https://classic.clinicaltrials.gov/ct2/show/NCT04620031).

**Supplementary Information:**

The online version contains supplementary material available at 10.1186/s13613-024-01390-3.

## Background

Currently, the most frequently administered sedative drugs to patients in the intensive care unit (ICU) are benzodiazepines, propofol and dexmedetomidine. The most commonly used benzodiazepine is midazolam which still has a very important role to play for deep sedation, seizure management or in combination with other analgesics/analgesics to reduce their respective adverse events (AEs) [1, 2]. Propofol reduces the mechanical ventilation duration and length of stays (LOS) in ICU compared to benzodiazepines because of its short-action, due to its large volume of distribution [3, 4]. Compared with benzodiazepines and propofol, dexmedetomidine significantly reduces the duration of mechanical ventilation and delirium incidence but also elicits a higher rate of bradycardia and hypotensive events [5, 6].

Regardless of the sedative agents used, the early ICU phase is also a crucial time for determining what depth of sedation to achieve and deep sedation tends to be accepted by many clinicians. However, this intensity of sedation often exposed patients to unnecessary prolonged deep sedation, where early deep sedation (48–72 h) was independently associated with prolonged extubation time and increased 6-month mortality [7]. This continued until 2013, when Shehabi et al. first introduced the concept of early goal-directed sedation (EGDS), in which patients treated with non-benzodiazepines reached light sedation goals within 12 h of endotracheal intubation or ICU admission, resulting in a shorter duration of mechanical ventilation and ICU LOS [8]. This confers the feasibility of light sedation as early as possible and 2013 guidelines for pain, agitation/sedation and delirium management also advocated the implementation of an EGDS strategy for critically ill patients to maintain light sedation unless they were in a state of acute stress or having unstable organ functions, for example patients suffering from acute respiratory distress syndrome [9]. Furthermore, a questionnaire survey conducted in the 2023 Annual Meeting of the Chinese Medical Association of Critical Care revealed that clinicians preferred to achieve rapid sedation targets without serious AEs (SAEs), with hypotension being the most concerning AE during sedation [10]. This also aligns with the Chinese guidelines on analgesic and sedative procedures, which recommend closely monitoring patients within the first 30 min after starting sedative medication [11]. Therefore, there is still a continuing clinical need to develop the clinical significance of achieving earlier light sedation (i.e., 30 min), so as to provide a basis for optimizing the sedation treatment regimen for ICU patients. The balance between sedation goals and sedation-related adverse effects (especially hypotension) during sedation in the ICU is noteworthy.

Ciprofol is an analogue of propofol but with a 4 ~ 5-fold increase in potency [12, 13], that was initially approved for clinical use in China in 2019. Ciprofol exerts its sedation/anesthesia effects through the activation of γ-aminobutyric acid type A (GABA_A_) receptors, and has a rapid onset of action and a rapid recovery time, lower rates of injection pain and less circulatory effects during intraoperative general anesthesia [14, 15], gastroscopy, colonoscopy and fiberoptic bronchoscopy [16–18]. Subsequently, ciprofol was approved for use in ICU patients requiring sedation during mechanical ventilation in July 2022, as both phase 2 and phase 3 clinical trials revealed that ciprofol had a favorable safety profile, with comparable sedation outcomes to that of propofol [19, 20]. However, the relatively small cohort sizes of the phase 2 and phase 3 trials limited the exploration of early sedation and its potential predictors for hypotension during short-period sedation. Therefore, data were pooled from patients in both trials to investigate hypotension-free outcome in ICU patients undergoing mechanical ventilation who achieved the target light sedation at an early stage (30 min) after dosing of ciprofol or propofol during a 6–24 h sedation period. Our hypothesis was that ciprofol treatment would resulted in a higher incidence of hypotension-free rates than propofol in patients who had achieved the light sedation goal at an early phase.

## Methods

### Study patients and interventions

The pooled analysis included the adult ICU patients (aged < 80 years) undergoing mechanical ventilation from phase 2 (November 2019 to May 2020) and phase 3 (December 2020 to June 2021), multi-center, randomized trials [19, 20]. All eligible patients were randomly allocated to a ciprofol or propofol group in a 2:1 ratio. In consideration of the safety risks for critically ill patients in the ICU, the phase 3 trial employed a single-blind design, where the research evaluators were blinded; patients and investigators were prohibited from exchanging information about the study drug. In addition, the phase 2 trial adopted an open-label design, the detailed randomizations and masking methods were presented in the Supplementary File 1. The phase 2 and phase 3 trial protocols were approved by First Affiliated Hospital of Sun Yat-sen University (phase 2 trial: 2019-037-02; phase 3 trial: 2020-057-03) and all other participating centers. The procedures used in this study adhered to the tenets of the Declaration of Helsinki and written informed consent was provided by all enrolled patients or a legal representative. The phase 2 trial (Clinicaltrials.gov identifier NCT04147416, Principal Investigator Xiangdong Guan, registered November 1, 2019) and phase 3 trial (Clinicaltrials.gov identifier NCT04620031, Principal Investigator Xiangdong Guan, registered November 6, 2020) were both prospectively registered with clinicaltrials.gov.

Patients were eligible to participate in the two clinical trials if they met the following criteria: a body mass index (BMI) ≥ 18 and ≤ 30 kg/m^2^; patients who required tracheal intubation and mechanical ventilation; individuals expected to require sedation for 6–24 h within the target light sedation range (RASS: -2 to + 1). Exclusion criteria for both trials included: patients who were known to be allergic to eggs, soybean products, opioid drugs and their rescue drugs or propofol; those who had contraindications to these chemicals; or a medical history of cardiovascular system, mental system disease, cognitive dysfunction, severe hepatic and renal insufficiency or dialysis at screening, which may have increased the risk of sedation/anesthesia. Patients with grand mal status epilepticus, craniocerebral injury, intracranial hypertension, cerebral aneurysm, high paraplegia or general paralysis or had an expected survival of ≤ 72 h; a Glasgow Coma Scale (GCS) ≤ 12 points; or a Sepsis-related Organ Failure Assessment (SOFA) > 9 points were also excluded. In addition, patients who had received sedation for more than 3 days in an ICU or in a general ward prior to being transferred to the ICU before signing an informed consent form were excluded from the phase 3 trial and patients who had the following laboratory indicators: neutrophil count ≤ 1.0 × 10^9^/L; platelet count ≤ 50 × 10^9^/L; hemoglobin ≤ 70 g/L) at screening were excluded from the phase 2 trial.

Both trials included screening, drug administration (6–24 h; loading and maintenance infusions) and follow-up periods. During the drug administration period of the phase 2 trial, ciprofol or propofol was initially infused for 0.5–5 min at a loading dose of 0.1–0.2 mg/kg or 0.5-1.0 mg/kg, followed by an initial maintenance dose rate of 0.3 mg/kg/h or 1.5 mg/kg/h, respectively. Of 39 patients in the phase 2 trial, only 5 were given a loading dose within 3 min, and the remaining dose was administered for more than 3 min. The pharmacokinetics/pharmacodynamics results revealed that ciprofol at a loading dose of 0.1 mg/kg given over 3–5 min, followed by an initial maintenance infusion rate of 0.2–0.3 mg/kg/h reached the 90% reference interval of the plasma concentration (55.6-212.6 ng/mL) within the target RASS range of -2 to + 1. Therefore, the loading dose regimen of the phase 3 trial changed to: ciprofol or propofol infused over 4 min ± 30 s at a loading dose of 0.1 mg/kg or 0.5 mg/kg, respectively. Moreover, the duration of the loading doses for ciprofol vs. propofol were 3.8 min vs. 3.9 min. In both trials, the dosage of ciprofol or propofol was adjusted up or down at a rate of 0.05–0.10 mg/kg/h or 0.25–0.50 mg/kg/h, respectively, in order to achieve target light sedation within the adjustment dose ranges of 0.06–0.80 mg/kg/h or 0.3-4.0 mg/kg/h, respectively. In both trials, if a patient required sedation prior to the study drug (ciprofol or propofol) administration, propofol was permitted to be administered intravenously at 0.25–0.50 mg/kg per dose, but the study drug administration was not initiated within 30 min after the last propofol administration. Moreover, patients were required to confirm that a RASS score of ≥ -2 was achieved at baseline sedation level before administration of the study drug could be initiated. Except for 1 assessment within 30 min prior to ciprofol/propofol dosing as a baseline value, RASS was evaluated every 30 ± 5 min from the start of ciprofol or propofol dosing until 1 h after dosing, every 2 h ± 10 min after 1 h of dosing until the end of dosing, and every 5 ± 1 min after the cessation of drug administration until RASS was ≥ 0. Remifentanil was administered if necessary at a loading dose of 0.5–1.0 µg/kg for analgesia prior to the initial administration of ciprofol or propofol to make sure that the Critical Care Pain Observation Tool (CPOT) was < 3 points. During the maintenance period, remifentanil was adjusted to a dose range of 0.02 ~ 0.15 µg/kg/min for analgesia when the CPOT was ≥ 3 points [21]. In addition, even if CPOT was < 3 points but patients complained of pain, the remifentanil dose was adjusted according to clinical judgement to reach the appropriate level of pain control.

## Outcomes and assessments

The primary post-hoc endpoint was the hypotension-free rate in patients who had achieved the target sedation goal after 30-min administration, defined as the proportion of patients who did not experience drug-related hypotension when at the required target light sedation level after 30 min administration of ciprofol or propofol. Hypotension was defined as a systolic blood pressure (SBP) < 90 mmHg or a 20% decrease from baseline, with a duration lasting more than 2 min [22].

The secondary efficacy endpoints included: the hypotension-free rate in patients who had achieved the target sedation goal after 1-h administration; sedation success rate; mean sedation compliance rate; time to extubation; and recovery time from sedation. The success of the sedation rate was the percentage of patients who met both of the following criteria: proportion of duration that RASS was in the range − 2 to + 1 was ≥ 70.0% of the total drug administration period [23]; and did not require rescue therapy. The mean sedation compliance rate was defined as the mean percentage of the duration that RASS was in the range − 2 to + 1 for the total study drug administration duration. Time to extubation was defined as time from ICU entry to extubation for patients intubated prior to ICU entry, or defined as the time from intubation to extubation for patients who were intubated after ICU admission. Recovery time from sedation was defined as the time from cessation of ciprofol or propofol administration to the patient being fully alert (RASS ≥ 0); recovery time was recorded as 0 if RASS was ≥ 0 at the time of drug discontinuation.

Safety evaluations included the incidence of treatment-emergent AEs (TEAEs), drug-related TEAEs (refer to TEAEs that were definitely related, probably related or possible related to the ciprofol/propofol treatment) and changes from baseline in SBP, diastolic blood pressure (DBP) and mean arterial pressure (MAP) after 30 min and 1 h administration of ciprofol or propofol. All TEAEs were classified according to the system organ class (SOC) and preferred term (PT) of the Medical Dictionary for Regulatory Activities (MedDRA), and the severity of a TEAE was graded in line with Common Terminology Criteria for Adverse Events (CTCAE) ver. 5.0 guidelines. Blood pressure (SBP, DBP and MAP) was monitored continuously throughout the drug administration period and recorded once within 30 min prior to ciprofol/propofol dosing as the baseline value, once every 30 ± 5 min from the start of ciprofol/propofol dosing to 1 h after administration, once every 1 h ± 10 min from 1 h after ciprofol/propofol dosing to the end of administration, and once within 2 h after the end of administration, and once during the follow-up period.

### Statistical analysis

All statistical analyses were performed using R software version 4.1.3 (R Foundation for Statistical Computing, Vienna, Austria). The stats package was used for univariate and multivariable logistic regression analyses as well as for Spearman’s rank correlation analysis. Additional packages utilized included dplyr for data manipulation and ggplot2 for data visualization. Continuous variables are presented as the mean ± standard deviation (SD) or maximum with range (minimum-maximum) and categorical variables as numbers with percentages. The Wilcoxon rank sum test was used to make comparisons between the two groups for non-normally distributed continuous variable, while a *t*-test was used for normally distributed continuous variables. Fisher’s exact test was employed to compare categorical variables with theoretical frequencies less than 5, while the chi-squared test was used for comparisons of other categorical variables. A two-sided test of *P* ≤ 0.05 was deemed to be a significant difference.

The efficacy outcomes were analyzed in the full analysis set (FAS), which included all patients treated with ciprofol or propofol after randomization, and had an available post-dose efficacy outcome. The safety set was comprised of patients who had been treated with ciprofol/propofol after randomization and had a post-dose available outcome, primarily for the analysis of TEAEs and blood pressure. The last observation carried forward (LOCF) approach was used to fill in the missing data of RASS during the drug administration, while the other data were not imputed. The exploratory subgroup analysis was carried out to investigate the primary post-hoc endpoint between two groups among the following subgroups: age, gender, BMI and SOFA classification. Univariate and multivariable logistic regression analyses were conducted to identify significant predictors for the primary post-hoc endpoint, in which indicators with a *P*-value < 0.1 in the univariate analysis were retained for multivariable logistic regression analysis. In addition, Spearman’s rank correlation analysis was carried out to explore the prognostic outcomes (i.e. drug-related TEAEs, time to extubation, recovery time from sedation, remifentanil dose/min per body weight and the number of dose adjustments for ciprofol and propofol) in hypotension-free patients who achieved the target light sedation after 30 min administration of ciprofol or propofol.

## Results

### Patient dispositions and baseline characteristics

**Fig. 1:**
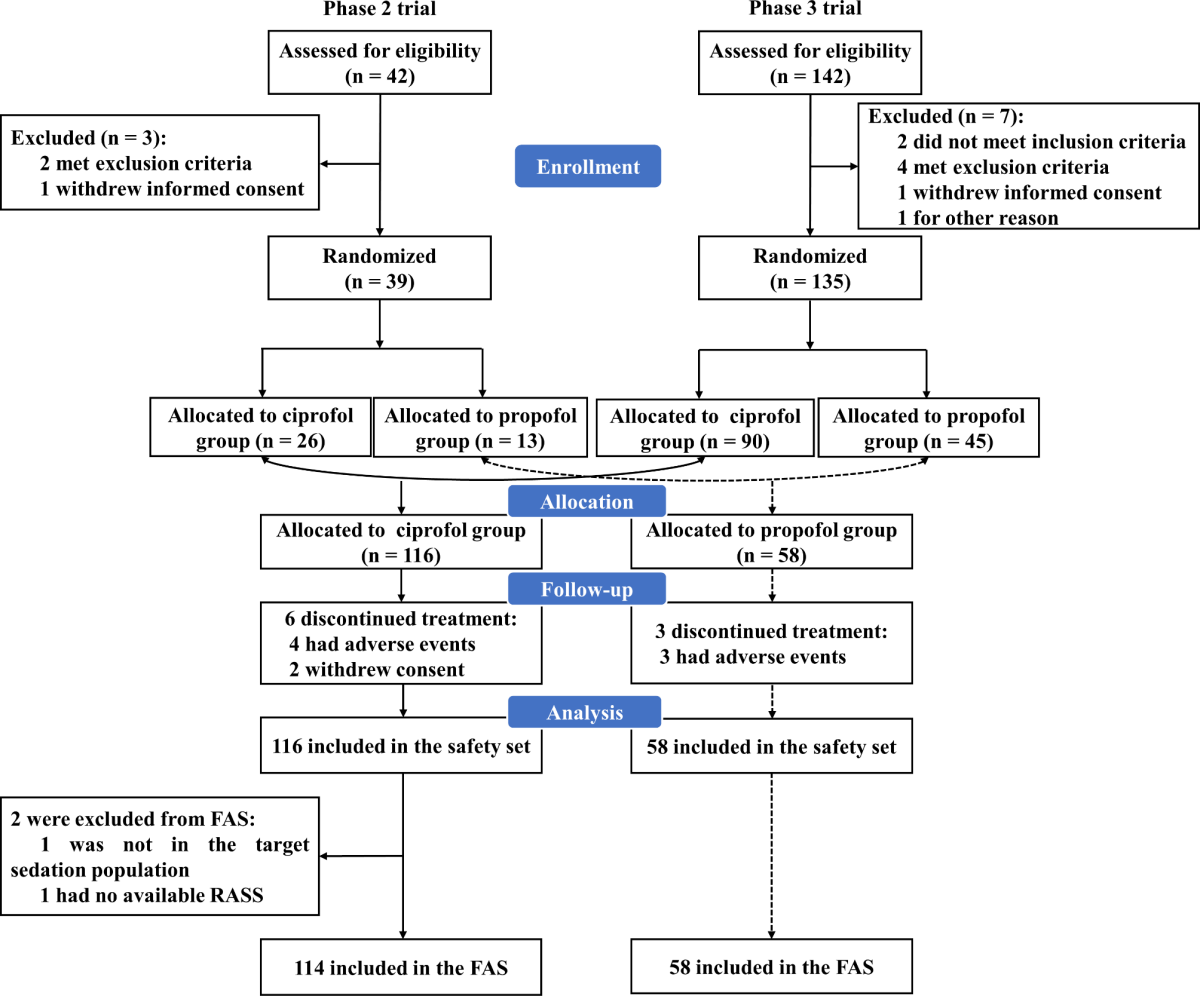
Patient disposition. Abbreviation FAS, full analysis set

There were 42 and 142 patients enrolled in the phase 2 and phase 3 clinical trials, respectively, of whom 39 and 135 were randomized (Fig. 1). A pooled analysis was conducted on 174 patients, of whom 116 and 58 were assigned to ciprofol or propofol groups, respectively. All 174 patients were enrolled in the safety set, of whom 172 were enrolled in the FAS. Most of the patients were younger than 65 years of age (69.3% vs. 81.0%). The occurrences of comorbidities were similar between the two groups, with hypertension (35.1% vs. 34.5%) and anemia (23.7% vs. 19.0%) being the most common (Table 1). Furthermore, the vast majority of patients in both groups were admitted to the ICU postoperatively (92.2% vs. 93.1%), with the most common operations in the ciprofol group being exploratory laparotomy (14, 12.3%), while lymphadenectomy (6, 10.3%) and uvulopalatopharyngoplasty (6, 10.3%) were predominant in the propofol group. Except for significant differences in baseline GCS (*P* = 0.028) and SOFA (*P* = 0.024), the remaining demographics and baseline characteristics were comparable between the two groups.

**Table 1:** Baseline characteristics and demographics of the enrolled patients

Items	Ciprofol (*n* = 114)	Propofol (*n* = 58)	*P*-value
Age (years)	55.91 ± 12.98	53.16 ± 12.73	0.187
Age distribution (years), n (%)			0.100
< 65	79 (69.3)	47 (81.0)	
≥ 65	35 (30.7)	11 (19.0)	
Gender, n (%)			0.810
Male	69 (60.5)	34 (58.6)	
Female	45 (39.5)	24 (41.4)	
BMI (kg/m^2^)	23.65 ± 3.18	23.72 ± 3.45	0.886
GCS (points)	15 (13, 15)	15 (14, 15)	0.028
SOFA (points)	1 (0, 6)	1 (0, 4)	0.024
Child-Pugh classification, n (%)			0.869
Class A	17 (14.9)	8 (13.8)	
Class B	8 (7.0)	3 (5.2)	
Not evaluable	89 (78.1)	47 (81.0)	
eGFR classification (mL/min/1.73 m^2^), n (%)			0.235
eGFR ≥ 90	87 (76.3)	49 (84.5)	
60 ≤ eGFR < 90	24 (21.1)	9 (15.5)	
30 < eGFR < 60	2 (1.8)	0	
eGFR ≤ 30	0	0	
Not evaluable	1 (0.9)	0	
Comorbidities, n (%)			
Hypertension	40 (35.1)	20 (34.5)	0.937
Anemia	27 (23.7)	11 (19.0)	0.480
Hepatic cyst	12 (10.5)	7 (12.1)	0.760
Infectious pneumonia	12 (10.5)	6 (10.3)	0.970
Renal cyst	11 (9.6)	5 (8.6)	0.826

Note Data are given as the mean ± SD, median with range (minimum, maximum) or numbers with percentages

Abbreviations BMI, body mass index; eGFR, estimated glomerular filtration rate; GCS, Glasgow coma scale; ICU, intensive care units; SOFA, sepsis-related organ failure assessment

### Efficacy outcomes

**Fig. 2:**
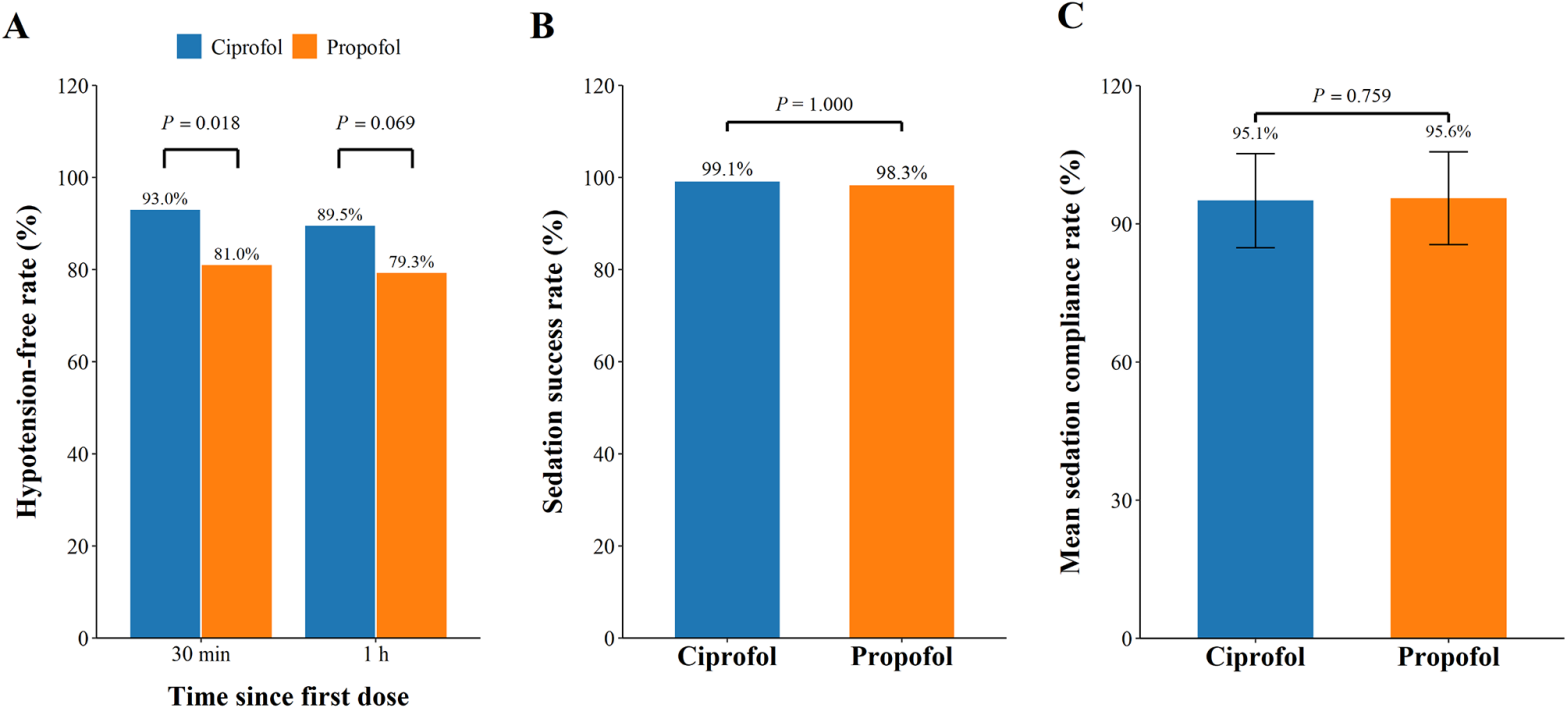
The pooled analysis of sedation-related rates in two groups. (**A**) The hypotension-free rate in the patients who achieved the sedation goal after 30 min and 1 h administration of ciprofol; (**B**) The sedation success rate and (**C**) mean sedation compliance rate during the overall drug administration period

There were 112 (98.2%) and 55 (94.8%) patients who achieved the light sedation goal after 30 min administration of ciprofol or propofol respectively, with drug-related hypotension occurring in 6 and 8 patients. Thus, patients treated with ciprofol had a significantly higher hypotension-free rate for those who achieved a target sedation goal after 30-min administration (93.0% vs. 81.0%, *P* = 0.018) (Fig. 2). An exploratory subgroup analysis showed that the hypotension-free rate in patients who had achieved the target sedation goal after 30-min administration was higher in the ciprofol group than in the propofol group for males (*P* = 0.019) and patients < 65 years of age (*P* = 0.027) (Table 2). Multivariable analysis revealed that ciprofol treatment (odds ratio [OR] 0.23, 95% confidence interval [CI]: 0.08–0.68; *P* = 0.008), young age (OR 0.91, 95% CI: 0.85–0.97, *P* = 0.003) and lower baseline BMI (OR 0.83, 95% CI: 0.70–0.99; *P* = 0.042) were significantly associated with a higher hypotension-free rate in patients who had achieved the target sedation goal after 30-min administration (Table 3). Moreover, hypotension-free patients who reached the target sedation level after 30 min administration exhibited lower rates of drug-related TEAEs (*r* = -0.412, *P* < 0.001), a shorter time to extubation (*r* = -0.250, *P* = 0.004) and fewer dose adjustments of ciprofol or propofol (*r* = -0.493, *P* < 0.001) (Table 4).

**Table 2:** Subgroup analysis of hypotension-free rate when achieved the target light sedation after 30 min administration between two groups (FAS)

Items	Hypotension-free rate when achieved the target light sedation after 30 min administration, *n*/*N* (%)		*P*-value
	Ciprofol (*n* = 114)	Propofol (*n* = 58)	
Age distribution (years)			
< 65	75/79 (94.9)	39/47 (83.0)	0.027
≥ 65	30/35 (85.7)	8/11 (72.7)	0.322
Gender			
Male	64/69 (92.8)	26/34 (76.5)	0.019
Female	41/45 (91.1)	21/24 (87.5)	0.636
BMI (kg/m^2^)			
< 24	66/69 (95.7)	28/31 (90.3)	0.299
≥ 24	39/45 (86.7)	19/27 (70.4)	0.091
SOFA classification (points)			
< 2	61/66 (92.4)	35/42 (83.3)	0.143
≥ 2	44/48 (91.7)	12/16 (75.0)	0.081

Note Data are given as numbers with percentages

Abbreviations BMI, body mass index; FAS, full analysis set; SOFA, sepsis-related organ failure assessment

**Table 3:** Univariate and multivariable logistic regression analyses of the hypotension-free rate when the target light sedation was achieved after 30 min administration (*n* = 172)

Indicators	Univariate analysis		Multivariable analysis	
	OR (95% CI)	*P*-value	OR (95% CI)	*P*-value
Treatment (ref: ciprofol)	0.40 (0.14–0.92)	0.034	0.23 (0.08–0.68)	0.008
Gender (ref: male)	1.28 (0.48–3.39)	0.620		
Baseline characteristics				
Age (years)	0.93 (0.89–0.98)	0.005	0.91 (0.85–0.97)	0.003
Height	1.03 (0.98–1.09)	0.257		
Weight (kg)	0.99 (0.95–1.03)	0.491		
BMI (kg/m^2^)	0.87 (0.76–1.01)	0.072	0.83 (0.70–0.99)	0.042
GCS (points)	0.43 (0.07–2.78)	0.374		
SOFA (points)	1.02 (0.74–1.41)	0.912		
eGFR (mL/min/1.73 m^2^)	1.02 (1.01–1.04)	0.008	1.02 (1.00-1.03)	0.097

Abbreviations BMI, body mass index; eGFR, estimated glomerular filtration rate; GCS, Glasgow coma scale; SOFA, sepsis-related organ failure assessment

**Table 4:** Spearman’s rank correlation analysis for prognostic outcomes in hypotension-free patients who reached the target sedation level after 30 min administration

Prognostic outcomes	*r* (Spearman)	*P*-value
Drug-related TEAEs	-0.412	< 0.001
Time to extubation	-0.250	0.002
Recovery time from sedation	0.026	0.741
Numbers of dose adjustments for ciprofol/propofol	-0.493	< 0.001
Remifentanil dose/min per body weight	0.024	0.755

Abbreviations ICU, intensive care units; TEAE, treatment-emergent adverse event

No significant differences were found between the ciprofol and propofol groups with regard to the hypotension-free rate in patients who had achieved the target sedation goal after 1 h administration (89.5% vs. 79.3%, *P* = 0.069), sedation success rate (99.1% vs. 98.3%, *P* = 1.000), or the mean sedation compliance rate (95.1 ± 10.2 vs. 95.6 ± 10.1 min, *P* = 0.759) (Fig. 2). In addition, the median values of time to extubation (1,015.0 vs. 1,022.5 min, *P* = 0.353), recovery time from sedation (0.1 vs. 0.0 min, *P* = 0.112), remifentanil unit dosage (0.04 vs. 0.04 µg/kg/min, *P* = 0.688), drug administration duration (8.8 vs. 8.6 h, *P* = 0.745) in the ciprofol group did not significantly differ from that in the propofol group (Table 5).

**Table 5:** Pooled analysis of sedation-related duration and remifentanil dosages in the two groups (FAS)

Items	Ciprofol (*n* = 114)	Propofol (*n* = 58)	*P*-value
Overall drug administration duration (h)	8.8 (1.6–24.0)	8.6 (2.3–21.9)	0.745
Time to extubation (min)	1015.0 (425–3535)	1022.5 (465–1697)	0.353
Recovery time from sedation (min)	0.1 (0–65.0)	0.0 (0-24.6)	0.112
Remifentanil dose/min per body weight (µg/kg/min)	0.04 (0.02-6.00)	0.04 (0.02–3.12)	0.688

Note Data are given as the mean ± standard deviation (SD) or median with range (minimum-maximum)

## Changes from baseline in blood pressures after 30 min and 1 h administration

The mean reduction changes in SBP, DBP and MAP from baseline after 30 min dosing of ciprofol or propofol were − 8.2 ± 17.7 vs. -12.6 ± 19.7 mmHg (SBP), -4.2 ± 10.3 vs. -6.2 ± 9.7 (DBP) and − 5.3 ± 11.3 vs. -8.4 ± 12.4 mmHg (MAP), respectively. However, these reduction changes from baseline after 30 min administration were not statistically significant (all *P* > 0.05) (see Supplemental Fig. 1). Similar results were also observed in patients after a 1 h administration of ciprofol or propofol.

## Treatment-emergent adverse events

There were 74 (63.8%) and 45 (77.6%) patients who experienced TEAEs, of which the majority of TEAEs were grade 1 or grade 2 in severity (Supplemental Table 1). Patients in the ciprofol group had a significantly lower rate of drug-related TEAEs (20.7% vs. 34.5%, *P* = 0.048). The most frequent drug-related TEAEs were hypotension (17.2% vs. 29.3%), bradycardia (2.6% vs. 3.4%), hypertriglyceridemia (2.6% vs. 1.7%) and respiratory depression (0.9% vs. 3.4%) in the ciprofol and propofol groups. The median duration was 90.0 vs. 138.5 min for drug-related hypotension, 120.0 vs. 435.5 min for drug-related bradycardia and 60.0 vs. 108.0 min for drug-related respiratory depression in patients treated with ciprofol or propofol, but statistical significance was not reached (all *P* > 0.05).

## Discussion

Previous studies have shown that achieving light sedation at an early stage leads to a more favorable prognosis, such as a shorter mechanical ventilation time [8, 24], whereas early deeper sedation and frequent hypotension both resulted in delayed extubation and an increase in long-term mortality [7, 25]. Accordingly, ciprofol/propofol sedation were used for light sedation, with RASS ranging from − 2 to + 1 in the present study, which was aligned with pain, agitation/sedation, delirium, immobility (rehabilitation/mobilization), and sleep (disruption) (PADIS) and Chinese guidelines [11, 26] to explore the risk factors for hypotension at the same depth of sedation. In the post-hoc analysis of pooled data from phase 2 and phase 3 trials, we mainly focused on the hypotension-free outcome of ICU patients undergoing mechanical ventilation who achieved the target light sedation at an early time (30 min) after dosing with ciprofol or propofol. Our main findings demonstrated that patients treated with ciprofol had a significantly higher incidence of hypotension-free rates when they achieved target light sedation after 30 min administration (93.0% vs. 81.0%, *P* = 0.018), especially in the subgroups of males and patients < 65 years old (all *P* < 0.05). As previously reported, ciprofol has also been shown to present a better hemodynamic profile, including less fluctuations in MAP, lower occurrences of hypotension and respiratory depression [27–29]. In addition to the ciprofol treatment, younger age and a lower baseline BMI were also independent favorable predictors for a higher hypotension-free rate when the target light sedation goal was reached after 30 min administration.

The Acute Physiology and Chronic Health Evaluation II (APCHE II) score serves as a reliable indicator of the severity of illness. Previous studies have shown that the baseline APACHE II score was a risk factor for hypotension after sedation in ICU patients, with patients with an APACHE II ≥ 10 points given larger doses of norepinephrine and for a longer duration than those with an APACHE II score < 10 points [25]. In the present study, the median APACHE II score (9.0 vs. 9.0 points, *P* = 0.078) at baseline was similar between the two groups in the phase 2 trial, while the baseline APACHE II data were not collected in the phase 3 trial, thus predictions for the APACHE II scores for hypotension were not available in the pooled analysis, which warrants investigation in a future long-term study. In addition, the baseline GCS and SOFA were both collected and even though there was an imbalance regarding the baseline GCS and SOFA between groups (all *P* < 0.05), there was no effect on the primary endpoint in either the subgroup analyses or the multifactorial analysis. It is worth noting that the majority of the patients included in this study were admitted to the ICU after surgery, and their baseline conditions were not severe and were comparable. In addition to the APACHE II score, impaired cardiovascular and liver function are both recognized as risk factors for hypotension after sedation [25]. However, this study did not include patients with severe hepatic and renal insufficiency, cardiovascular diseases, craniocerebral injury, as well as other critically ill patients with an expected survival time of less than 72 h, as the potential benefit for these individuals remained uncertain.

Clinically, many patients often experience hypotension after sedation, leading to tissue hypoperfusion and deterioration of organ functions, which is not conducive to postoperative recovery, and postoperative hypotension also resulted in an increased risk of 30-day major adverse cardiac or cerebrovascular events and a higher 30-day mortality in critically ill patients [30]. These results suggested that for severely ill patients, maintaining a lower level of sedation within the range of light sedation while achieving the sedation goal may be advantageous in reducing the probability of hypotension occurrence or shortening the duration of hypotension, thus improving patient prognosis. In the study, patients who reached the target sedation level after 30 min administration also presented with a better short-term prognosis (within 24 h), including lower rates of drug-related TEAEs, shorter time to extubation and fewer dose adjustments of ciprofol/propofol. Another issue with sedation was the occurrence of delirium and studies have shown that postoperative hypotension after sedation was associated with an increased risk of postoperative delirium in severely ill patients [31]. In the phase 2 trial, delirium assessments were conducted before the initial administration of ciprofol/propofol and within 2 h after the end of administration, and negative results of Confusion Assessment Method for the Intensive Care Unit (CAM-ICU) were reported. It is pity that the occurrence of delirium was not set as the endpoint in the phase 3 trial, so that the limitation of the pooled analysis was that prognosis correlations between hypotension after sedation and delirium were not available. Overall, this pooled analysis explored the potential predictions and prognosis of hypotension-free outcomes within a short-term period (within 24 h) in ICU patients undergoing mechanical ventilation. We excluding patients with moderate to severe hepatic or renal dysfunction, as well as other critically ill patients with an expected survival time of < 72 h, which lacks broad generalizability and requires further validation in a broader population and to establish long-term prognostic outcomes.

Additionally, ciprofol has been demonstrated to have comparable sedation profiles with regard to the sedation success rate, mean sedation compliance rate, time to extubation (all *P* > 0.05), as previously reported in two clinical trials [19, 20]. In contrast, fewer drug-related TEAEs occurred in patients in the ciprofol group compared to the propofol group, whereas the recovery time from sedation was comparable [19], which may be attributed to the pooled analysis having a larger sample size, thus reinforcing the evidence that ciprofol elicited fewer side effects, which is in good agreement with a meta-analysis of ciprofol in non-ICU sedation [32]. The present study employed a sedation strategy prioritizing analgesia so that a CPOT < 3 points was ensured for each patient before the initial sedation with ciprofol/propofol. The dose of remifentanil was adjusted to maintain the CPOT < 3 points during the maintenance period so that the changes in CPOT before and after sedation were minimal between the two groups. Patients receiving ciprofol did not exhibit a significant difference in the dose of remifentanil (0.04 vs. 0.04 µg/kg/min, *P* = 0.688) compared to those receiving propofol after pooled analysis of the two trials, a finding in good agreement with previous ciprofol studies [14, 19, 20]. Moreover, Spearman’s rank correlation analysis also indicated that there was no correlation between the occurrence of hypotension and the dosage of remifentanil in patients who achieved the target light sedation goal.

The limitations of this study were that it was a post-hoc analysis from two trials with insufficient statistical power. The patients enrolled were those projected to require a short sedation period of 6 to 24 h, with a median sedation time of 8.8 h and 8.6 h in the ciprofol and propofol groups, respectively. Thus, potential long-term complications (over 24 h) of ciprofol in ICU patients were not observed in the present study, and warrants further investigation. Indeed, an exploratory study aimed at investigating the long-term sedation effects of ciprofol in ICU patients receiving mechanical ventilation for up to 96 h has been initiated (NCT04669821).

## Conclusions

A continuous infusion of ciprofol significantly improved the hypotension-free rate in patients who had achieved the target light sedation at an early time, resulting in a favorable short-term prognosis for ICU patients undergoing mechanical ventilation during a 6–24 h sedation period, without new safety findings of concern being found. The predictions and prognosis for hypotension-free outcomes should be interpreted with caution, as the trials excluded those with moderate to severe hepatic or renal dysfunction, as well as other critically ill patients with an expected survival time of < 72 h.

## Electronic supplementary material

Below is the link to the electronic supplementary material.

Supplementary Material 1

## Data Availability

The datasets used and/or analysed during the current study are available from the corresponding author on reasonable request.

## Abbreviations

AE: Adverse event
APCHE II: Acute Physiology and Chronic Health Evaluation II
BMI: Body mass index
CAM-ICU: Confusion Assessment Method for the Intensive Care Unit
CPOT: Critical Care Pain Observation Tool
CTCAE: Common Terminology Criteria for Adverse Events
DBP: Diastolic blood pressure
EGDS: Early goal-directed sedation
FAS: Full analysis set
GABA_A_: γ-aminobutyric acid type A
GCS: Glasgow Coma Scale
ICU: Intensive care unit
LOCF: Last observation carried forward
LOS: Length of stays
MAP: Mean arterial pressure
MedDRA: Medical Dictionary for Regulatory Activities
OR: Odds ratio
PADIS: Pain, agitation/sedation, delirium, immobility (rehabilitation/mobilization), and sleep (disruption)
PT: Preferred term
RASS: Richmond Agitation-Sedation Scale
SAE: Serious adverse event
SBP: Systolic blood pressure
SD: Standard deviation
SOC: System organ class
SOFA: Sepsis-related Organ Failure Assessment
TEAE: Treatment-emergent adverse event
