# Supplementary material for: Early sedation using ciprofol for intensive care unit patients requiring mechanical ventilation: a pooled post-hoc analysis of data from phase 2 and phase 3 trials
Source: Ann Intensive Care. 2024 Oct 26;14:164. doi: 10.1186/s13613-024-01390-3 (PMC11511798; doi:10.1186/s13613-024-01390-3)
Supplement: Supplementary file 1 — Supplementary Material 1 [file 13613_2024_1390_MOESM1_ESM.docx]

**Supplementary Material**

**Supplementary File 1. Randomizations and masking**

***Phase 2 trial***

The phase 2 trial adopted an open-label design, a random number and a corresponding drug number for eligible patients was generated by a central random system, based on the interactive web response system.

***Phase 3 trial***

Before initiation of the study, an independent investigator who had no contact with any participant used SAS 9.4 software (SPSS Inc., USA) to generate a blocked random number table (block size = 5) to divide eligible patients in a 2:1 ratio into either a ciprofol or propofol groups (without stratification). The central randomization method (Interactive Web Response System) was employed across 21 centers to generate the random number and the corresponding drug number using Medidata system (Medidata, France).

Due to the differences in dose adjustment between ciprofol and propofol during the drug administration period, it was difficult to blind the investigators. Considering the safety risks for seriously ill patients in the ICU, a single-blind design was adopted for the present trial, in which the study evaluators were set as blind; both patients and investigators were not allowed to communicate with each other about study drug information. Moreover, the blind study evaluators were mainly responsible for assessing the patient's pain and sedation levels and then decided on when to start drug administration, dose adjustment trends and the time to the end of drug administration. They provided timely corresponding information to the non-blind study investigator who actually implemented the dose adjustments procedures, to determine the dosage and drug administration according to the grouping. The study investigators (non-blind) were required to calculate the initial administration dose and the top-up doses of patients in advance before drug administration. When giving the drug to patients in ICU, the study investigator could not disclose the administration dose, such as ‘‘initial administration of 5 mg,’’ but only said ‘‘initial administration,’’ ‘‘maintenance administration,’’ “start of injection’’ or ‘‘end of administration.’

**Supplemental Table 1. Summary of adverse events**

|  | **Ciprofol (n = 116)** | **Propofol (n = 58)** | ***P*-value** |
| --- | --- | --- | --- |
| Any TEAE, n (%) | 74 (63.8) | 45 (77.6) | 0.065 |
| Grade 1 | 60 (51.7) | 34 (58.6) |  |
| Grade 2 | 36 (31.0) | 22 (37.9) |  |
| ≥ Grade 3 | 13 (11.2) | 9 (15.5) |  |
| Any SAE, n (%) | 3 (2.6) | 2 (3.4) | 1.000 |
| Drug-related TEAEs, n (%) | 24 (20.7) | 20 (34.5) | 0.048 |
| Hypotension | 20 (17.2) | 17 (29.3) | 0.067 |
| Bradycardia | 3 (2.6) | 2 (3.4) | 1.000 |
| Hypertriglyceridemia | 3 (2.6) | 1 (1.7) | 1.000 |
| Respiratory depression | 1 (0.9) | 2 (3.4) | 0.258 |
| Atrial fibrillation | 1 (0.9) | 0 | 1.000 |
| Allergic dermatitis | 1 (0.9) | 0 | 1.000 |
| Elevated hemobilirubin | 0 | 1 (1.7) | 0.333 |
| Duration of drug-related TEAEs (min) | | | |
| Hypotension | 90.0 (5.0-714.0) | 138.5 (15.0-10,899.0) | 0.191 |
| Bradycardia | 120.0 (50.0-344.0) | 435.5 (151.0-720.0) | 0.248 |
| Respiratory depression | 60.0（60.0-60.0） | 108.0 (96.0-120.0) | 0.221 |

Note. Data are given as the median with range (minimum-maximum) or numbers with percentages.

Abbreviation. TEAE, treatment-emergent adverse event


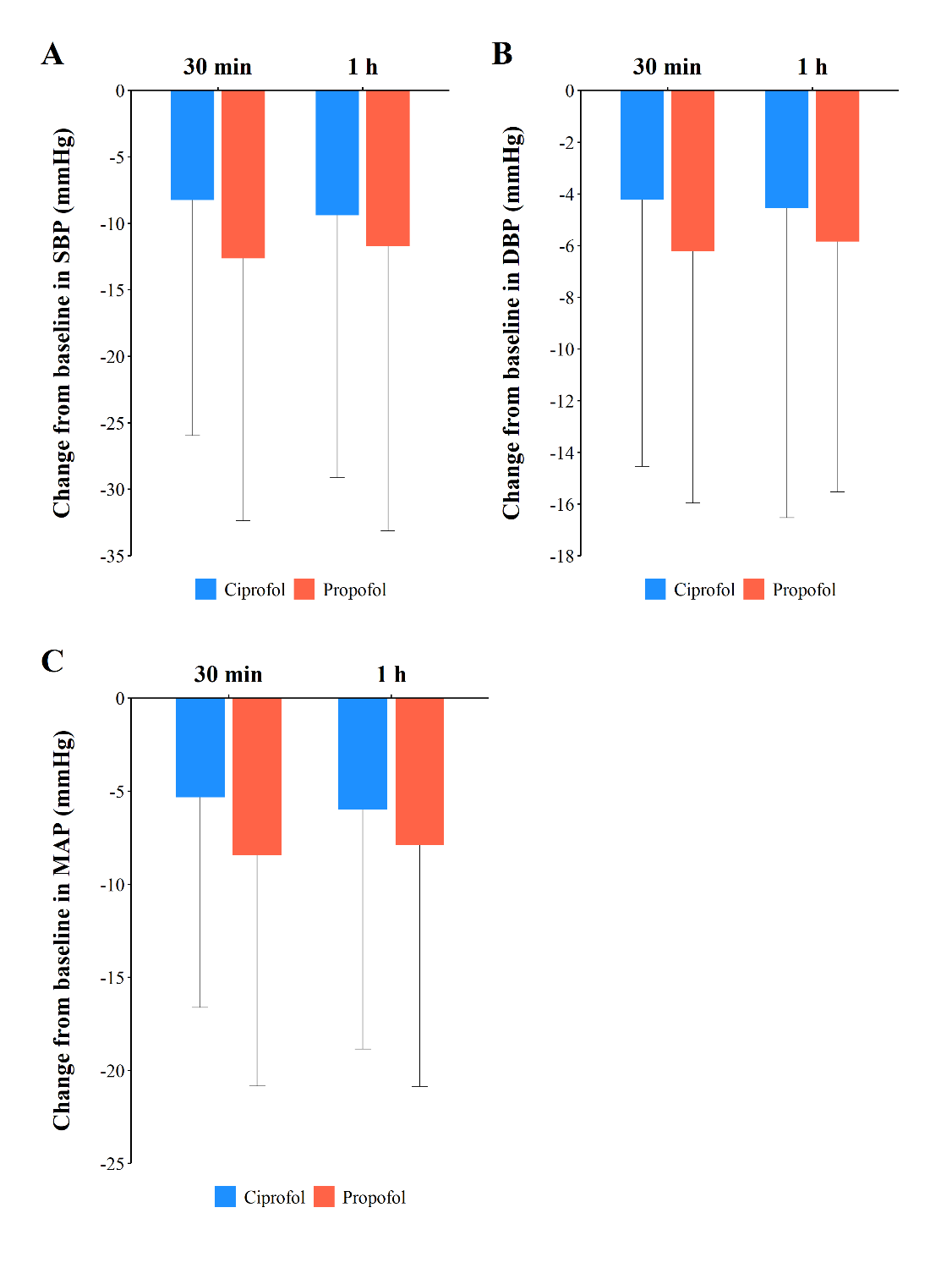
**Supplemental Fig. 1. Blood pressure changes from baseline after 30 min and 1 h administration of ciprofol or propofol between groups**

(A) Systolic blood pressure (SBP), (B) diastolic blood pressure (DBP) and (C) mean arterial pressure (MAP). Data are presented as the mean ± standard deviation (SD).
